# Apoptotic Activity of MeCP2 Is Enhanced by C-Terminal Truncating Mutations

**DOI:** 10.1371/journal.pone.0159632

**Published:** 2016-07-21

**Authors:** Alison A. Williams, Vera J. Mehler, Christina Mueller, Fernando Vonhoff, Robin White, Carsten Duch

**Affiliations:** 1 School of Life Sciences, Arizona State University, Tempe, Arizona, United States of America; 2 Institute of Zoology- Neurobiology, Johannes Gutenberg University Mainz, Mainz, Germany; 3 Institute of Physiology, University Medical Center, Mainz, Germany; 4 Molecular, Cellular, and Developmental Biology Department, Yale University, New Haven, Connecticut, United States of America; University of Insubria, ITALY

## Abstract

Methyl-CpG binding protein 2 (MeCP2) is a widely abundant, multifunctional protein most highly expressed in post-mitotic neurons. Mutations causing Rett syndrome and related neurodevelopmental disorders have been identified along the entire *MECP2* locus, but symptoms vary depending on mutation type and location. C-terminal mutations are prevalent, but little is known about the function of the MeCP2 C-terminus. We employ the genetic efficiency of *Drosophila* to provide evidence that expression of p.Arg294* (more commonly identified as *R294X*), a human *MECP2* E2 mutant allele causing truncation of the C-terminal domains, promotes apoptosis of identified neurons *in vivo*. We confirm this novel finding in HEK293T cells and then use *Drosophila* to map the region critical for neuronal apoptosis to a small sequence at the end of the C-terminal domain. *In vitro* studies in mammalian systems previously indicated a role of the MeCP2 E2 isoform in apoptosis, which is facilitated by phosphorylation at serine 80 (S80) and decreased by interactions with the forkhead protein FoxG1. We confirm the roles of S80 phosphorylation and forkhead domain transcription factors in affecting MeCP2-induced apoptosis in *Drosophila in vivo*, thus indicating mechanistic conservation between flies and mammalian cells. Our findings are consistent with a model in which C- and N-terminal interactions are required for healthy function of MeCP2.

## Introduction

Methyl-CpG binding protein 2 (MeCP2) is a ubiquitous and multifunctional protein most highly expressed by mature neurons [1]. Both MeCP2 loss- and gain- of function cause neurodevelopmental disorders, Rett Syndrome or *MECP2* Duplication Syndrome, respectively, which are characterized by severe cognitive, language, and motor impairments. While mutations causing Rett Syndrome and related disorders have been identified across the entire length of the *MECP2* locus [2], the severity and range of symptoms varies between patients depending on the location and nature of the *MECP2* mutation [3–6]. Accordingly, analysis of the various molecular and cellular functions of different domains of MeCP2 will be a useful basis to better understand MeCP2-related pathophysiology.

MeCP2 is traditionally known as a transcriptional repressor that binds to methylated CpG regions via the methyl binding domain (MBD) and silences local gene expression via the transcription domain (TRD) [7]. However, MeCP2 has additionally been shown to activate transcription in mouse models [8] and can form complexes with RNA binding proteins [9]. MeCP2 can also bind non-methylated DNA [10, 11], and such interactions may influence local chromatin structure [10].

On the cellular level, MeCP2 mis-regulation negatively impacts dendritic structure as shown in patients [12], *in vivo* in mouse [13–16], *Xenopus* [17], and *Drosophila* [18, 19] models, as well as in primary neuron and slice cultures [20, 21]. Additionally, overexpression of *MECP2*, specifically the E2 isoform, contributes to apoptosis in cultured neurons [22, 23]. In cerebellar granule neurons, MeCP2-induced apoptosis is inhibited by elevated levels of the forkhead protein FoxG1, mutations of which also cause Rett-related diseases. However, the molecular mechanisms of MeCP2-induced apoptosis and the relationship to MeCP2-related pathophysiology are not well understood.

Here, we utilize a *Drosophila* MeCP2 gain-of-function model to investigate the role of MeCP2 in neuronal cell death *in vivo*. Facile genetic tools [24], short generation time, and a high degree of conservation in the molecular mechanisms underlying apoptosis [25–27] make *Drosophila* a useful model for studying MeCP2-related cell death. While there is no *Drosophila MECP2* ortholog, multiple MeCP2 interactors and most components of the chromatin machinery are conserved in the fly. When expressed in *Drosophila*, human MeCP2 will associate with chromatin, modify transcription, and become phosphorylated at the same sites as in mammals [28]. Furthermore, expressing human *MECP2* in the fly has helped identify novel MeCP2 interactors that have been subsequently validated in mouse model systems [19, 28].

Using this model, we provide evidence that MeCP2 promotes apoptosis *in vivo* and identify a role for the C-terminus and serine 80 phosphorylation in mediating this effect. We show that MeCP2 gain-of-function apoptosis in *Drosophila* is likely acting via the same cellular pathways as in mammalian cells, and have established a behavioral assay that can be used for high-throughput screening to identify additional molecular players in MeCP2 induced neuronal apoptosis. Paradoxically, disease prognosis in patients carrying C-terminal truncations is less severe than for other MeCP2 mutations [4, 29–31]. We speculate that apoptosis caused by MeCP2 truncation during early brain development may ultimately result in a high proportion of healthy neurons with the mutation carrying X-chromosome inactivated.

## Materials and Methods

### Drosophila stocks

*Drosophila melanogaster* were reared in 68 ml vials on a standard yeast corn meal diet at 25°C and 60% humidity with a 12 hour light/dark cycle. All previously generated and new *MECP2* variants were derived from *MECP2*-E2 isoform cDNA. Previously generated p-element insertion lines UAS-*MECP2FL* and UAS-*R294X* [28] were kindly provided by Dr. J Botas (Baylor College of Medicine, Houston, Texas). CD8:PARP::VENUS flies were kindly provided by Dr. J. Truman (HHMI Janelia Research Institute, Ashburn, Virginia). Microinjection of all constructed pUASTattB vectors into embryos carrying the attP2 landing site (BDSC Stock # 8622) was done by Bestgene (www.thebestgene.com/). For cellular analyses, we used the C380-GAL4 driver which selectively expresses UAS-transgenes in a subset of motoneurons and other unidentified neurons [32, 33]. For cylinder drop experiments, the Cha-GAL80 transgene was included to inhibit expression in unidentified cholinergic sensory neurons and interneurons thus eliminating most known pre-synaptic connections [32]. ELAV(C155)-GAL4 was used to drive pan neuronal expression of UAS-transgenes for protein analysis [34]. GAL-4 driver lines were crossed to W1118 flies to generate genetic non-MECP2 carrying controls for all experiments.

#### Drosophila genotypes

**Fig 1:**
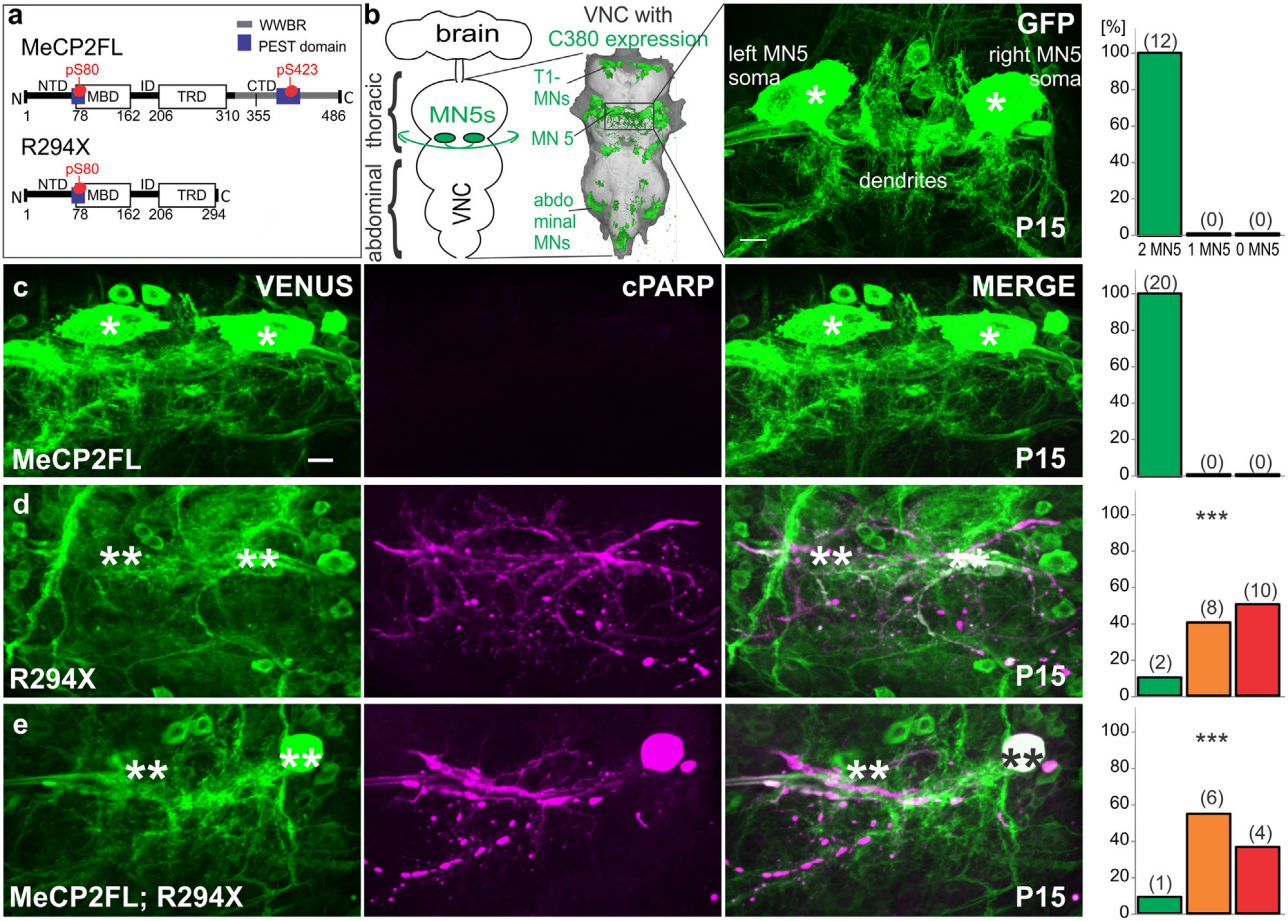
R294X truncation causes caspase mediated apoptosis in *Drosophila* MN5. a. Structural schematics of the *MECP2FL* and *R294X* alleles used for experiments. Only the *MECP2* E2 isoform is shown and was used for experiments. b. Schematic of *Drosophila* central nervous system showing localization of motoneuron 5 (MN5) within the ventral nerve cord (VNC). GFP labeling with the C380-GAL4 motoneuron driver line is shown overlaying the VNC, and confocal projections views of GFP labeled MN5 somata are shown as selective enlargements at right. c-e. Representative images of flies at pupal stage P15 expressing either *MECP2FL* (c), *R294X* (d), or both alleles (e) under the control of C380-GAL4. Histograms show the percentage of preparations with either 0, 1, or 2 MN5 somata present for each genotype. Exact number of flies corresponding to each bar is reported in parentheses. Both MN5 somata and no cPARP reactivity was found with control (b) or *MECP2FL* (c). Most MN5 somata expressing either *R294X* alone (d) or together with *MECP2FL* (e) were missing, and high cPARP reactivity was found in the remaining somata and neuronal projections. The distribution of intact MN5s in (d) and (e) significantly differed compared to expression of *MECP2FL* alone (c) (*** *p* < 0.0001, Pearson’s chi-square). Single asterisks indicate intact MN5 somata, double asterisks indicate absent or severely deteriorated somata. Scale bar depicts 10 μm.

Fig 1b: C380-GAL4, UAS-mcd8-GFP/X;;Cha-GAL80/+ (control)

1c: C380-GAL4/X;;UAS-MECP2FL/UAS-mcd8-hPARP:VENUS

1d: C380-GAL4/X;;UAS-R294X/UAS-mcd8-hPARP:VENUS

1e: C380-GAL4/X;UAS-MECP2FL/+;UAS-R294X/UAS-mcd8-hPARP:VENUS

Fig 2: none

Fig 3a: C380-GAL4/X;;UAS-R294X/UAS-mcd8-hPARP:VENUS

3b: C380-GAL4/X;;UAS-V312X/UAS-mcd8-hPARP:VENUS

3c: C380-GAL4/X;;UAS-K431X/UAS-mcd8-hPARP:VENUS

3d. C380-GAL4/X;;UAS-V481X/UAS-mcd8-hPARP:VENUS

3e. C380-GAL4/X;;UAS-MECP2FL/UAS-mcd8-hPARP:VENUS

Fig 4a: C380-GAL4/X;;UAS-MECP2FL/UAS-mcd8-hPARP:VENUS

4b: C380-GAL4/X;;UAS-MECP2FLS80E/UAS-mcd8-hPARP:VENUS

4c: C380-GAL4/X;;UAS-R294XS80A/UAS-mcd8-hPARP:VENUS

Fig 5a: C380-GAL4/X;;UAS-R294X/UAS-mcd8-hPARP:VENUS

5b: C380-GAL4/X;UAS-slp1;UAS-R294X/UAS-mcd8-hPARP:VENUS

5d, e:

Control: C380-GAL4, UAS-mcd8GFP/Y;;cha-GAL80/+

MECP2FL: C380-GAL4, UAS-mcd8GFP/Y;;UAS-MECP2FL/cha-GAL80

R294X: C380-GAL4, UAS-mcd8GFP/Y;;UAS-R294X/cha-GAL80

Slp1;R294X:: C380-GAL4, UAS-mcd8GFP/Y;UAS-slp1/+;UAS-R294X/cha-GAL80

S1 Fig: (all): C380-GAL4/X;;UAS-R294X/UAS-mcd8-hPARP:VENUS

S2 Fig: C380-GAL4/X;;UAS-MECP2FL/UAS-mcd8-hPARP:VENUS

C380-GAL4/X;;UAS-V481X/UAS-mcd8-hPARP:VENUS

C380-GAL4/X;;UAS-K431X/UAS-mcd8-hPARP:VENUS

C380-GAL4/X;;UAS-V312X/UAS-mcd8-hPARP:VENUS

C380-GAL4/X;;UAS-R294X/UAS-mcd8-hPARP:VENUS

S2b Fig: ELAV(C155)-GAL4/Y;;UAS-MECP2FL/+

ELAV(C155)-GAL4/Y;;UAS-K431X/+

**Fig 2:**
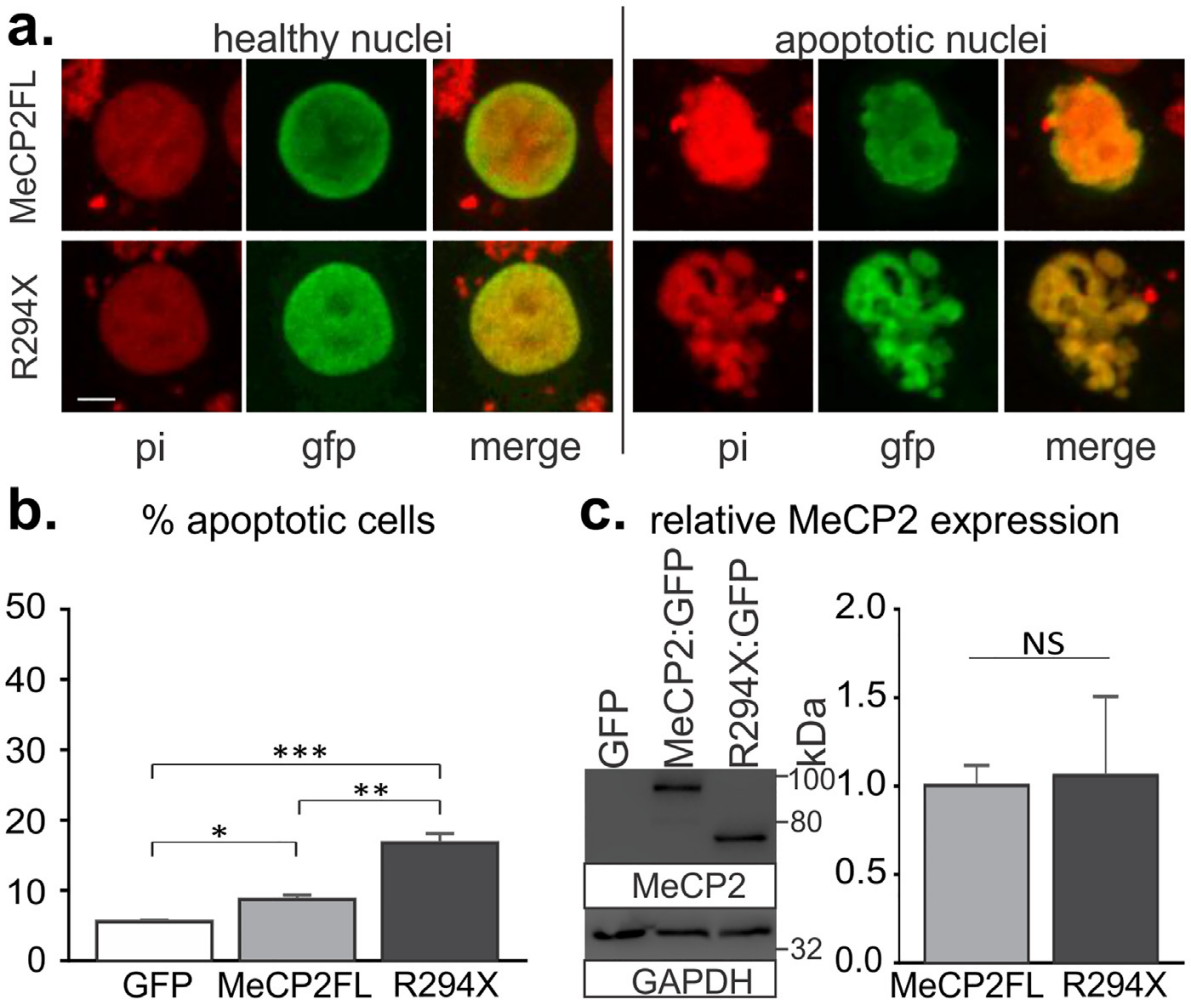
*R294X* transfection promotes cell death in mammalian cell culture to a higher degree than *MECP2FL*. a. Representative images of HEK293T cells transfected with GFP tagged *MECP2FL* or *R294X*. Examples of healthy transfected cells are at left, while the condensed and/or fragmented nuclei at right were counted as apoptotic. b. Quantification of apoptotic cells following transfection of *MECP2FL*:*GFP*, *R294X*:*GFP*, or GFP control. n = 6 independent transfections/group, with > 400 cells counted for each independent transfection. Percentage data was transformed using the arcsine square root function to meet the assumptions for a one way ANOVA (F(2,15) = 46.81, p < .0001). * p < .05, ** p < .01, *** p < .001 Tukey post-hoc test. c. Relative *MECP2* expression 24 hours following transfection into HEK293T cells. No differences were observed in relative protein levels at 24 or 48 hours post-transfection (Mann-Whitney U test). Scale bar depicts 5μm. pi = propidium iodide, NS = not significant.

**Fig 3:**
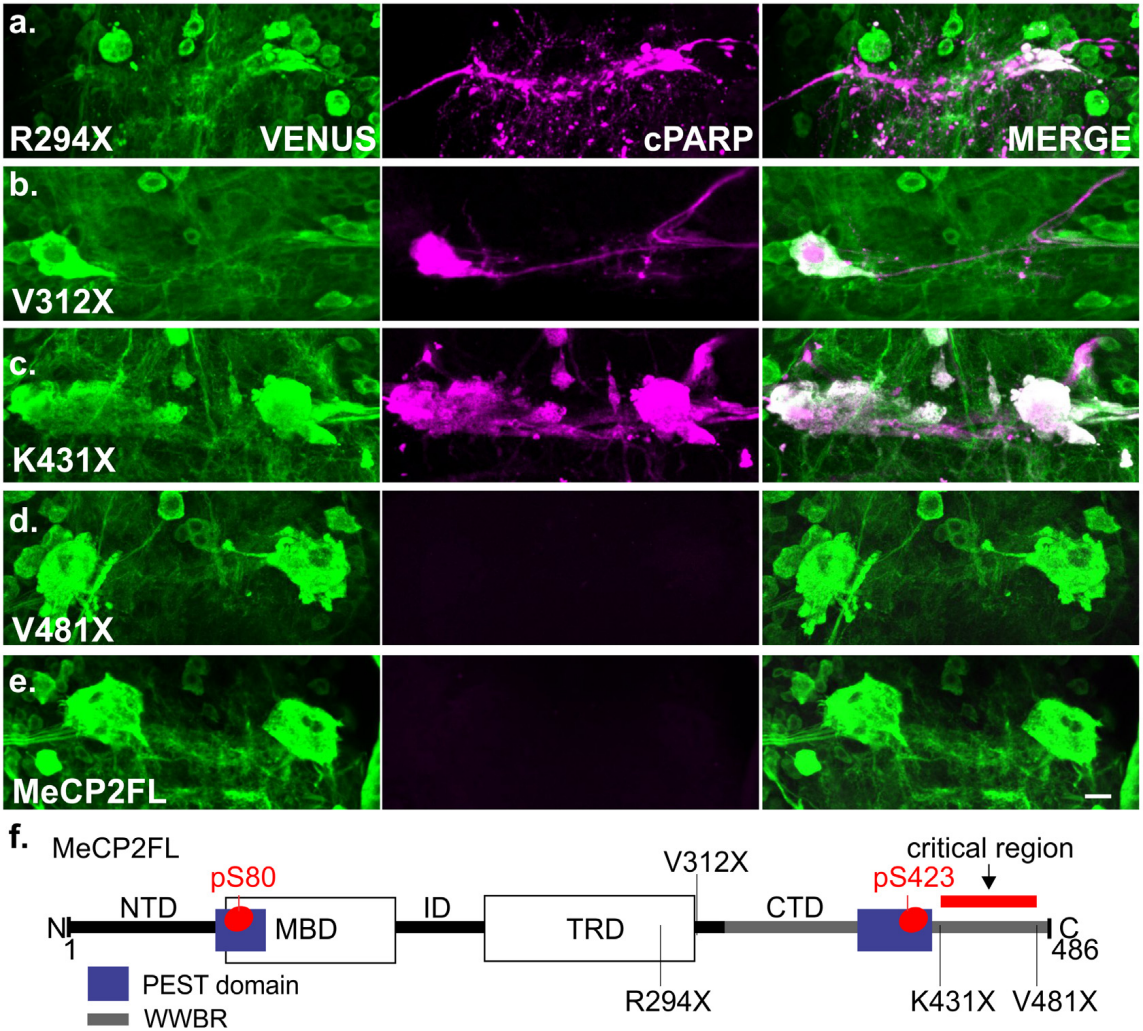
MeCP2 C-terminal domain is critical for preventing apoptosis in Drosophila MN5. a-e. Representative images of cPARP reporter activity in MN5 from flies expressing C-terminal truncated variants of *MECP2* at pupal stage P15. All c-terminal truncations except V481X (d) caused caspase mediated apoptosis in MN5. f. Schematic of *MECP2* E2 isoform with mapped truncations. The region between AA431-481 was found to be critical in preventing apoptosis as caused by truncated MeCP2 in MN5. Scale bar depicts 10 μm.

**Fig 4:**
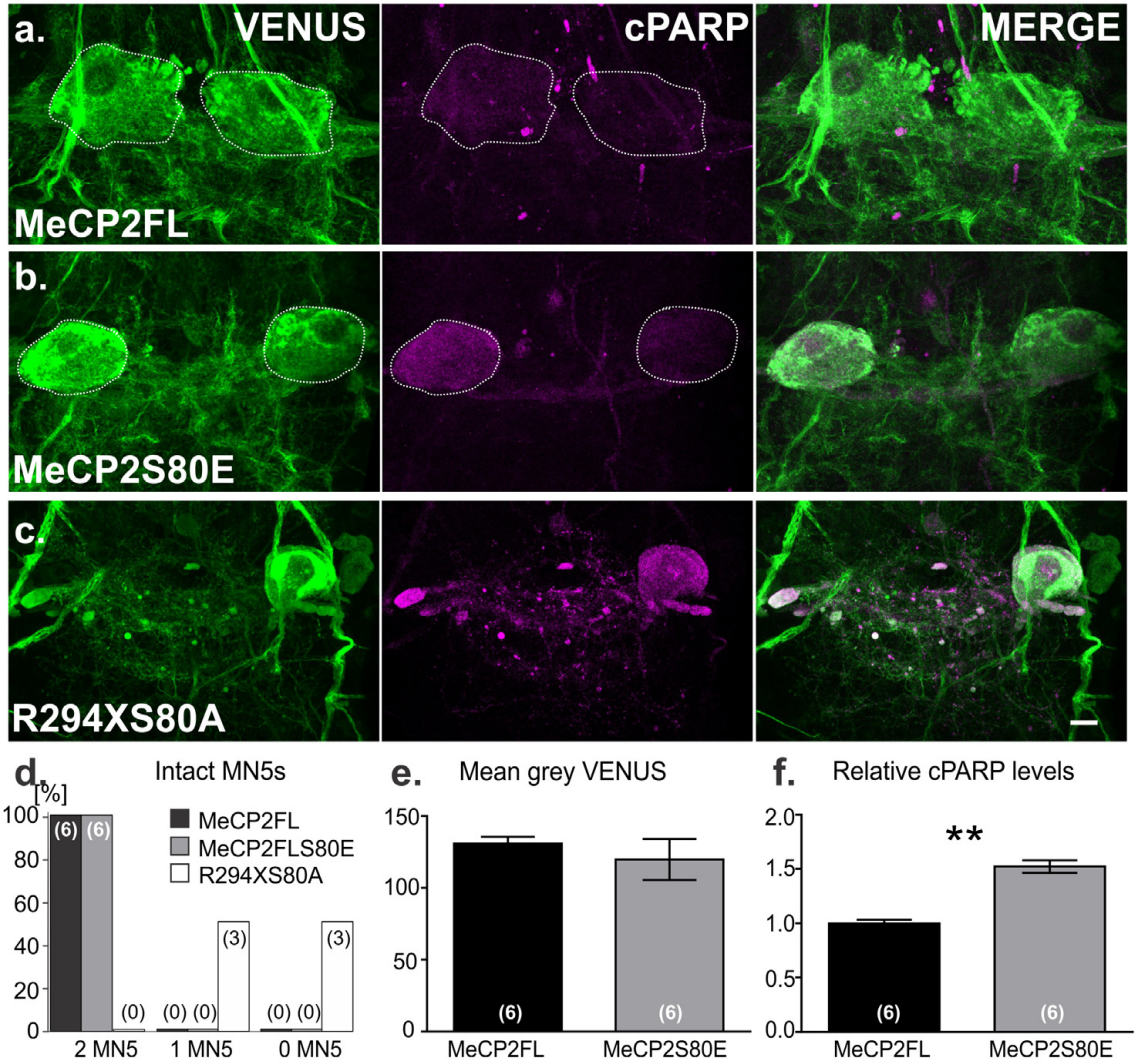
S80 phosphorylation mediates caspase activity in full-length but not truncated MeCP2. a-c. Representative images of cPARP reporter activity in MN5. Intact MN5 cell bodies are outlined in white. b. Phosphomimicking mutation S80E increases caspase activity in MN5 compared to controls (a), while phosphoblocking mutation S80A (c) has no effect on the toxicity of the R294X truncation. d. Percentage of preparations examined 0–24 hours post-eclosion with either 0, 1, or both MN5s (numbers in bars indicate number of animals with the respective phenotype) e-f. Quantification of caspase activity visualized by immunocytochemistry. Individual MN5 somata (white dashed lines in a-b) were traced and mean grey values were calculated using ImageJ. Expression of *MECP2FLS80E* increased cPARP reactivity in comparison to expression of normal *MECP2FL* (f). No differences in VENUS reactivity were observed (e). ** p <0.005, Pearson’s chi-square (d) or Student’s two-tailed t-test (f). Six flies from two independent crosses were observed and analyzed for each genotype. Scale bar depicts 10 μm. Error bars show mean +/- SEM.

**Fig 5:**
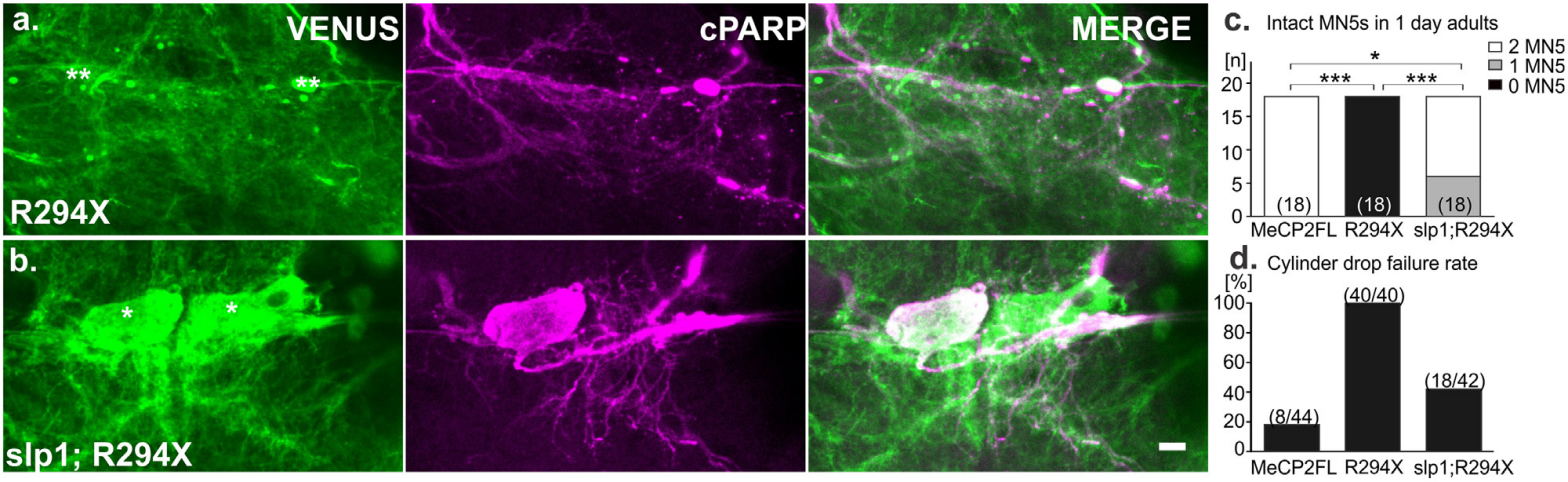
Co-expression of *slp1* improves cellular and behavioral consequences of R294X at one day post-eclosion. a-b. Representative images of cPARP reactivity in MN5s at 1–2 days post-eclosion. Single asterisks mark intact MN5s, while double asterisks denote absent MN5s. c. Distribution of preparations with 0, 1, or both MN5s is presented for flies expressing *MECP2FL*, *R294X*, or co-expression of *slp1; R294X* (* p < 0.05, *** p < 0.0001 Pearson’s chi square). d. Percentage of flies failing to initiate flight in the cylinder drop test. All flies tested were collected from at least three independent crosses for each genotype. N’s are overlaid in parentheses on histogram bars for each experimental group. Scale bar depicts 10μm.

### Molecular biology

The pUASTattB vector was kindly provided by the lab of Dr. K. Basler (Institute of Molecular Life Sciences, Zurich, Switzerland). Human *MECP2*-E2 cDNA was obtained from the DNASU Plasmid Repository at Arizona State University (Clone ID HsCD00434196). *MECP2FLS80E* and S80A plasmids were previously generated [23] and kindly provided by Dr. C. Kilstrup-Nielsen (University of Insubria, Busto Arsizio, Italy). To generate new full-length and truncated *MECP2* constructs, PCR amplified sequences were cloned into pUASTattB using *EcoRI* and *NotI* (New England Biolabs). Truncated constructs were generated by inserting a TGA stop codon prior to the 3’ NotI site. The following primers were used to amplify and generate new pUASTattB constructs:

FWD (all constructs): 5’ GAATTCCCACCATGGTAGCTGGGAT 3’

MECP2FL/MECP2FLS80E REV: 5’ GAGGAGCGGCCGCTCAGCTAACTCTCTCGGTCACGG 3’

R294X/R294XS80A REV: 5’ GAGGAGCGGCCGCTCAGATAGAAGACTCCTTCACGGCTT 3’

V312X REV: 5’ GAGGAGCGGCCGCTCACGTCTCCCGGGTCTTGC 3’

L431X REV: 5’ GAGGAGCGGCCGCTCAGGGGCAGCCGTCG 3’

V481X REV: 5’ GAGGAGCGGCCGCTCAGGGCGTCCGGCTGTCCAC 3’

For transfection and expression in cell culture, PCR amplified sequences were cloned into the pN3GFP plasmid at the *EcoRI* and *BamI* sites using the following primers:

FWD: 5’ GAATTCCCACCATGGTAGCTGGGAT 3’

MECP2FL REV: 5’ GAGGAGGATCCGCTAACTCTCTCGGTCACGG 3’

R294X REV: 5‘ GAGGAGGATCCGATAGAAGACTCCTTCACGGCTT 3‘

### Cell culture

HEK293T cells were kindly provided by Dr. C. Pietrzik (University Medical Center Mainz, Germany) and cultured in DMEM+Glutamax (Life technologies) supplemented with 10% fetal calf serum and 10000U Penicillin/Streptomycin (Life technologies) at 37°C and 5%CO_2_. Cells were regularly tested for microbial contamination (including mycoplasma). Per glass coverslip, 30000 cells were plated and transfected using 1μl Fugene HD (Promega) with 0.75μg Plasmid DNA according to manufacturer’s protocol. Some coverslips were treated with 2μM staurosporine for 18 h to serve as a positive control for apoptotic morphology.

### Western blotting

For cell culture experiments, cells were harvested by scraping in cold lysis buffer (50mM Tris, 150 mM NaCl, 1mM EDTA, 1% Triton-X) containing cOmplete EDTA-free protease and PhoStop phosphatase inhibitors (Roche). For fly experiments, whole adult *Drosophila* heads (10 per sample) were dissected directly into cold lysis buffer. Proteins were separated by SDS-PAGE and transferred onto nitrocellulose (Karl Roth) membranes. Membranes were blocked in 4% milk in TBST (0.05M Tris, 0.15 M NaCl, pH7.2, 0.1% (v/v) Tween20) for 30 minutes at room temperature and primary antibodies were applied overnight at 4°C in blocking medium. GAPDH [35] (rabbit; Bethyl Laboratories A300-641A) and MeCP2 [28] (rabbit; Thermo Scientific PA1-887) antibodies were both used at 1:5,000 for Western blot. Anti-HSP90 [36] (rabbit, Cell Signaling Technology #4874) was used at 1:1,000. Anti-rabbit horseradish peroxidase (Dianova #111-035-144, 1:10,000) was applied for one hour at room temperature.

### Immunohistochemistry

*Drosophila* were dissected and ganglia were fixed in 4% paraformaldehyde (PFA) in PBS for 1 hour and washed in PBS (0.1M). Tissue was permeated with 0.5% Triton X in PBS (6 X 30 minute washes), and primary antibodies and secondary antibodies were applied overnight at 4°C in 0.3% Triton X in PBS or in PBS, respectively. Preparations were washed in PBS, progressively dehydrated in an ascending ethanol series, and mounted in methyl salicylate. Cell cultures were fixed for 15 minutes in 4% PFA in PBS on coverslips and washed in PBS. Cells were permeated with 0.1% Triton X in PBS for two minutes, washed in PBS and incubated with primary antibodies in PBS for 1–2 hours at room temperature or overnight at 4°C. Secondary antibodies were applied for 30–60 minutes and coverslips were mounted onto glass slides with Fluoromount™ (Sigma-Aldrich). For propidium iodide staining [37], cells were first treated with 0.1 mg/ml RNase (Sigma-Aldrich) for 15 minutes at 37°C followed by 50 μl of a 500 μM propidium iodide stock solution (Sigma-Aldrich) for 30 minutes at room temperature.

Stacks of 0.5 μm (*Drosophila* whole mount) or 1.0 μm (cell culture) optical sections with 1024 X 1024 resolution were acquired on a Leica TCS SP8 laser-scanning confocal microscope (LSCM) with a 20X (0.75 NA) or 40X (1.2 NA) oil-immersion lens.

### Antibodies

The following primary antibodies were used for cell culture and/or whole mount stainings: Rabbit anti-cleaved PARP Ab2317 [38] (1:500, Abcam), chicken anti-GFP A10262 [39] (1:400, Life Technologies), and mouse anti-MeCP2 ab55538 [40] (1:400, Abcam). Rabbit cleaved caspase-3 ASP175 [35] (1:100, Cell Signaling Technology) was used to confirm apoptotic nuclei (data not shown). All secondary antibodies, including goat anti-chicken Alexa 488 (Life Technologies A-11039), donkey anti-Mouse Alexa 568 (Life Technologies A10037), donkey anti-mouse Alexa 647 (Jackson Immuno Research 715-605-150), and donkey anti-rabbit Cy5 (Jackson ImmunoResearch 711-175-152) were applied at a concentration of 1:400.

### Image analysis

For HEK293T cell experiments, transfection was confirmed by GFP immunohistochemistry and cell viability was assessed by propidium iodide staining, with condensed or fragmented nuclei counted as apoptotic, as described by others [22, 41, 42]. Each transfection experiment was replicated six times, with at least 400 cells counted per transfection.

For quantitative analysis of cPARP immunoreactivity in MN5, all preparations analyzed were subjected simultaneously to the identical immunohistochemistry procedure by processing them in the same dish, and images were acquired with the exact same LSCM parameters within one imaging session. To analyze images, the mean grey value of each MN5 somata was calculated for the cPARP channel using Image J. This value was normalized to the respective grey value measured from the GFP channel to control for potential differences in reporter availability.

### Flight assay

The “cylinder drop assay” was adapted with minor modifications from a previously described flight test [43]. Briefly, male flies were collected by suctioning at 24 hours post-eclosion and distributed into 0.5ml Eppendorf tubes with a small filter paper containing 5μl a 10% sucrose solution at the bottom. After a two hour acclimation period, flies were injected individually into a 33.3cm glass cylinder using a custom made spring-loaded releasing device. The landing height of each fly was recorded, and any non-flyers were collected at the bottom of the cylinder.

### Statistical analysis

FoxG1/slp1 sequence alignment was conducted using Jalview Version 2.9 [44]. Statistical analyses were conducted using GraphPad Prism 6.0 or R Statistical Software. Non parametric statistics were used in the event a data set did not meet the assumptions of normality or equal variance between groups. Graphical representations were prepared using GraphPad Prism 6.07 and CorelDraw X7.

## Results

### R294X gain-of-function causes apoptosis in MN5

Mutations causing Rett syndrome have been identified in all annotated functional domains of *MECP2* but occur most abundantly in the two best characterized domains, the MBD (AA 78–162) and the TRD (AA 207–310) [45] (Fig 1a). In a mouse model of *MECP2* duplication syndrome, both the MBD and TRD are required for the behavioral consequences of MeCP2 gain-of-function [46]. We have previously shown that the dendritic phenotype observed with human *MECP2* expression in an identified *Drosophila* neuron, motoneuron 5 (MN5), is also dependent on an intact MBD [18]. MN5 is a monopolar flight motoneuron that innervates the major wing depressor muscle [36, 47]. MN5 is well suited for analyzing genetic interactions and cellular consequences of *MECP2* gain-of-function alleles because it is (i) individually identifiable, (ii) displays a stereotyped and well quantified morphology [48], (iii) is physiologically well described [49–52], and (iv) can be addressed by targeted genetic manipulation [18, 19, 53, 54] with the binary GAL4-UAS expression system [55]. We use the C380-Gal4; Cha-Gal80 driver line to heterologously express human *MECP2* in MN5 labeled with GFP [18, 19, 48, 49, 53, 54]. C380-GAL4 expresses in about 30 neurons per hemisegment of the *Drosophila* ventral nerve cord (VNC), most of which are glutamatergic motoneurons including MN5 (Fig 1b) [33]. Both left and right MN5 can easily be visualized in the mesothoracic neuromere by GFP expression (Fig 1b).

Using this model, we sought to examine the role of the TRD with gain-of-function in *Drosophila* by expressing the truncated human *MECP2* allele p.Arg294* (*R294X*), which accounts for about 5% of typical Rett patients [56], in MN5. To our surprise, the large, readily identifiable somata of MN5 on both sides of the mesothoracic neuromere (Fig 1b) were missing in most adult flies expressing *R294X*, a phenomenon not observed with expression of full-length *MECP2* (*MECP2FL*) or MBD mutated *MECP2*. While we have previously found that expression of *MECP2FL* reduces MN5 dendritic length and branching, somata are not affected and have normal membrane currents [18]. Quantification at one day post-eclosion showed that MN5 somata were present on both sides of the VNC in 100% of all control and *MECP2FL* expressing flies (Fig 1b and 1c). Following expression of *R294X*, both MN5 somata were present in only one of twenty flies (Fig 1d). Of the remaining flies, only one MN5 was present in 40% of preparations and both MN5 somata were missing in the remaining 50%, yielding a significantly different distribution compared to *MECP2FL* alone (Pearson’s chi-square = 32.73, *p* < 0.0001) (Fig 1d). To determine whether this was due to a developmental defect or post mitotic cell death we examined the ventral nerve cords at various pupal stages and always found MN5 somata on both sides of the VNC up to pupal stage P8 (about 50% of pupal life) and occasionally up to pupal stage 15 (S1 Fig).

To establish whether the cell death occurred due to caspase-mediated apoptosis or another form of neuronal toxicity, we co-expressed a genetically encoded caspase activity reporter CD8::hPARP::Venus [38]. With this reporter, activated *Drosophila* caspases will cleave the human PARP protein tethered to a membrane bound Venus tag, and caspase activity can be visualized using an antibody specific to human cleaved PARP (cPARP). Following expression of *R294X* we detected caspase activity throughout residual degenerating arborizations of MN5 even after the somata had already disappeared at early adult stages (Fig 1d). By contrast, following expression of *MECP2FL* we could not detect significant levels of caspase activity in the somata or any arbors of MN5 (Fig 1c). C380-GAL4 drives expression of UAS-transgenes from early pupal stage P5 through adulthood [54]. During pupal life, caspase activity could first be detected with *R294X* expression in MN5 at stage P10 and increased at later pupal stages (S1 Fig) indicating the effects of *R294X* were not immediate upon onset of transgene expression.

Between P10 and eclosion, cell death patterning and timing is variable between flies, but no intact cell bodies were found beyond one day post-eclosion in over 30 flies analyzed. We further determined that the effects of R294X exert a dominant negative effect over full-length, intact MeCP2, as concomitant expression of *R294X* with *MECP2FL* led to high caspase activity and cell death in MN5 in a similar pattern as *R294X* alone (Fig 1e). Analysis of the distribution of remaining MN5’s at one-day post-eclosion revealed a significant difference between flies co-expressing *R294X* and *MECP2FL* compared to *MECP2FL* alone (Pearson’s chi-square = 28.84, *p* <0.0001) but no difference compared to *R294X* alone (Pearson’s chi-square = 0.6307, *p* = 0.7295).

### R294X gain-of-function increases MECP2 induced apoptosis in cell culture

While increased expression of *MECP2* has been shown to enhance apoptosis in various cell lines and in primary neuron cultures [22, 23], effects of truncated MeCP2 have not been directly investigated in mammalian systems. To test whether R294X contributes to apoptosis in a system with endogenous *MECP2* expression, we transfected HEK293T cells with GFP-tagged R294X, MeCP2FL, or control vector. Consistent with previously published data [22, 23], overexpression of *MECP2FL* increased the number of apoptotic cells as compared to GFP-transfected controls (Fig 2a and 2b). Transfection with *R294X*, however, led to a significantly higher increase in cell death despite similar protein expression levels (Fig 2a–2c). *R294X* expression therefore causes apoptosis in both *Drosophila* motoneurons and cultured HEK293 T cells.

### Apoptosis as caused by C-terminal truncation is not mediated by a known functional domain

Upon establishing validity of the *Drosophila* model system, we sought to use this system to determine which region of the missing 192 amino acids accounts for the critical phenotypic difference between R294X and MeCP2FL. While the entire C-terminal domain (CTD) is missing in the *R294X* allele, this mutation also disrupts the TRD by deleting the last 16 amino acids. Additionally, a proline-rich WW domain binding region (WWBR) has been identified spanning much of the CTD, from AA 325–486 [57]. While this domain can directly interact with the WW domains of RNA binding proteins, its function is not well understood [57]. In addition to S80, MeCP2 has activity-dependent phosphorylation sites located in the CTD at S421 and S424 of the murine E2 isoform [20, 58], corresponding to S423 and S426 of human MeCP2-E2 [59]. Both phosphorylation sites are located within one of two predicted PEST domains [60], which are generally associated with rapid proteolytic degradation [61]. To test whether the apoptotic effect was mediated by one of these known functional regions of MeCP2, we generated transgenic flies expressing three newly engineered C-terminal truncations: p.Val312*/V312X (with intact TRD but truncated CTD), p.Lys431*/K431X (truncation beyond the predicted C-terminal PEST domain), and p.Val312*/V481X (truncation eliminating the last five amino acids of the WWBR). New flies, including also new *MECP2FL* and *R294X* transgenes, were made using PhiC31 mediated site specific integration into the Attp2 landing site on chromosome III [62] to eliminate the possibility of positional effects due to transgene insertion site. All new constructs expressed in MN5 under the control of the C380-GAL4 driver with proper nuclear localization (S2a Fig).

To test whether the new truncated variants caused apoptosis in MN5, 10 flies for each genotype were dissected and stained for cPARP at pupal stage P15. We observed caspase activity and cell death in MN5 in all flies with expression of *R294X*, *V312X* and *K431X* (Fig 3a–3c), but none with *V481X* or *MECP2FL* (Fig 3d and 3e). This suggests that the CTD between amino acids 431–481 accounts for the critical difference in cell death (Fig 3f). Thus apoptosis cannot be rescued by replacement of intact TRD or PEST domains, and an intact WWBR is not required for MN5 survival. We found no differences in relative MeCP2 protein levels with quantitative Western blots from fly heads following pan neuronal expression of either *MECP2FL* or the *K431X* variant (S2b Fig). This indicates that the observed effects are unlikely caused by variable protein levels.

### Serine 80 phosphorylation contributes to cell death in vivo

In cell culture, phosphorylation at S80 has been shown to contribute to MeCP2-related apoptosis [23]. Specifically, blocking S80 phosphorylation by an alanine substitution (S80A) reduces apoptosis while a phosphomimicking glutamate substitution (S80E) leads to increased apoptosis [23]. To determine whether MeCP2 S80 phosphorylation contributes to apoptosis *in vivo*, we generated a transgenic fly line expressing the phosphomimicking S80E substitution (*MECP2FLS80E*) as UAS-transgene. Expression of *MECP2FLS80E* increased cPARP reactivity, indicative of higher caspase activity, in adult MN5s at one day post-eclosion compared to normal *MECP2FL* (Fig 4a and 4b). Quantification from 6 animals of each group revealed that, following expression of *MECP2FLS80E*, caspase activity in MN5 somata was significantly increased in comparison to expression of *MECP2FL* as a control (Fig 4e and 4f). However, although the phosphomimicking S80E substitution increased caspase activity, its effect on MN5 was less severe than C-terminal truncations. Consequently, in all 6 animals tested MN5 somata of adult flies at 0–1 day post-eclosion with MeCP2FLS80E were always present on both sides of the VNC (Fig 4d), whereas R294X caused most MN5 somata to die by this stage (Fig 1d).

We further designed a new *R294X* allele with the S80A substitution to test whether phosphorylation at S80 is required for apoptosis as caused by C-terminal MeCP2 truncation. As with expression of unaltered *R294X* (Figs 1d and 3a), cPARP reactivity was observed in all preparations with expression of *R294XS80A* (Fig 4c). At one day post-eclosion, at least one MN5 somata was missing in all flies (Fig 4d), yielding a significantly different distribution compared to *MECP2FL* (Pearson’s chi-square = 12, *p* = 0.0025). Thus, while MeCP2 S80 phosphorylation likely contributes to apoptosis, or at least caspase activation, C-terminal truncations cause apoptosis independent of S80 phosphorylation status (see discussion).

### Co-expression of Slp1 delays apoptosis and improves motor behavior

In cultured mammalian neurons, promotion of neuronal death by the full-length mouse *Mecp2*-E2 isoform is inhibited by forkhead protein FoxG1, and increasing FoxG1 can inhibit toxicity caused by increased Mecp2 [22]. FoxG1 directly binds Mecp2 via a 20 amino acid sequence in its DNA binding domain [22], a region that is highly conserved in the *Drosophila* FoxG1 ortholog *slp1* (S3 Fig). To determine whether cell death in *Drosophila* MN5 as caused by human R294X may be modified by the same mechanism, we co-expressed *R294X* with *slp1* and assessed MN5 viability at one day post-eclosion. In comparison to *R294X* expression alone, which eliminated both MN5s in all preparations, all flies co-expressing *slp1* displayed at least one (33% of preparations), and more often both, MN5 somata at 1–2 days post eclosion (67% of preparations). However, even in flies with both intact MN5s cPARP immunoreactivity revealed high caspase activity throughout the somata and neuronal arborizations in either one or both MN5s at this time point (Fig 5a and 5b). This suggests that slp1 only partially rescues the apoptotic effect of R294X. Statistical comparison of the number of MN5s present in all flies at 1–2 days post-eclosion revealed a significant rescue with *slp1* co-expression in comparison to *R294X* alone (Pearson’s chi-square = 36.00, p < 0.0001, Fig 5c). The *slp1;R294X* group was still statistically different from those with expression of *MECP2FL* (Pearson’s chi-square = 7.2, p = 0.0073, Fig 5c), thus supporting the qualitative partial rescue observed with the cPARP reporter (Fig 5a and 5b).

We then tested whether the slp1 mediated partial rescue of R294X induced motoneuron death improved motor function. We used a well-established cylinder drop test [43, 63, 64] to test whether flies were able to initiate flight and land on the wall of a glass cylinder before falling 33 cm to the bottom of the cylinder. While not a single fly out of 40 expressing *R294X* alone were able to initiate flight, 57% of all flies co-expressing *slp1* could fly (Fig 5d). In accordance with our data on the cellular level, motor behavioral performance was only partially rescued. Although co-expression of *slp1* with *R294X* significantly improved flight initiation over *R294X* alone, the *slp1;R294X* group performed significantly worse as compared to flies expressing *MECP2FL* (Fig 5d). Therefore, performance in this simple behavioral assay reflects previously identified genetic interaction and cellular phenotypes. This indicates that this tool may be useful in high-throughput screening with the multiple genome wide transgenic available *Drosophila* libraries for future identification of novel players involved in the apoptotic function of MeCP2 (see discussion).

## Discussion

### Drosophila as a model system to study the role of MeCP2 in apoptosis

In this study, we used a *Drosophila* model of MeCP2 gain-of-function to identify a critical role for the MeCP2 CTD in cell survival. Our data indicate that C-terminal truncating mutations of *MECP2* cause neuronal apoptosis *in vivo*. Given that *Drosophila* lacks a common *MECP2* ortholog and shows scarce genomic methylation [65] it is clear that this model will not recapitulate all aspects of MeCP2 related pathophysiology. However, multiple lines of reasoning suggest that our novel findings in *Drosophila* are relevant to understanding the cellular consequences of MeCP2 mutations in mammalian brains. First, we replicate our findings in HEK293T cell culture to confirm that C-terminal truncation causes cell death in a mammalian system. Expression of the C-terminal truncation *R294X* significantly increases the apoptotic effect of the E2 isoform of full-length MeCP2 that was previously reported in cultured mammalian neurons [22]. Second, in *Drosophila*, co-expression of the *FoxG1* ortholog *slp1* decreases the toxicity observed with *MECP2* C-terminal truncations *in vivo*. FoxG1 is a known inhibitor of MeCP2 toxicity in primary mammalian neuron cultures [22] and mutations in FoxG1 can also cause Rett related diseases [66–70]. Third, the apoptotic potential of full-length MeCP2 in *Drosophila* motoneurons is enhanced by serine 80 phosphorylation, as has been reported for numerous mammalian cell lines [23]. Together, our data suggest that C-terminal mutations can cause *MECP2* to induce apoptosis, and that this specific function is conserved in *Drosophila*. While apoptosis has not been reported *in vivo* with MeCP2 C-terminal truncation or gain-of-function mouse models, *MECP2* overexpression in chicken increases cell death in the developing neural tube [71]. However, it will be important to further validate our findings *in vivo* in mammalian neurons with C-terminal truncating mutations of the endogenous MeCP2 locus.

Generally, the molecular mechanisms underlying apoptosis are highly conserved from nematodes and insects to mammals [25–27], and MeCP2-induced apoptosis in fly motoneurons can accordingly be ameliorated by co-expression of *slp1*, the *Drosophila* ortholog of mammalian *FoxG1*. We show that this genetic interaction can be detected with a simple behavioral screen that can easily be modified for high-throughput screening. This, combined with facile tools for genetic manipulation and the availability of transgenic and mutant fly lines for almost all genes [24], renders *Drosophila* a useful tool to identify additional factors involved in MeCP2-related apoptosis.

### MeCP2 C- to N-terminal interactions are likely required for healthy MeCP2 function

We found that truncations of the MeCP2 C-terminus cause apoptosis in *Drosophila* MN5, and enhance the apoptotic role of the MeCP2 E2 isoform in HEK293T cells. In *Drosophila*, this effect is replicated with truncated variants up to K431X, which eliminates only 55 amino acids of the CTD leaving intact TRD and PEST domains. Cells expressing the short V481X truncation, eliminating the last five amino acids of the WWBR, however, remain healthy. These data indicate that the CTD region between AA431-481 is critical for preventing MeCP2 related apoptosis, but this is not caused by the loss of a critical health-promoting function of an identified functional domain.

**Fig 6:**
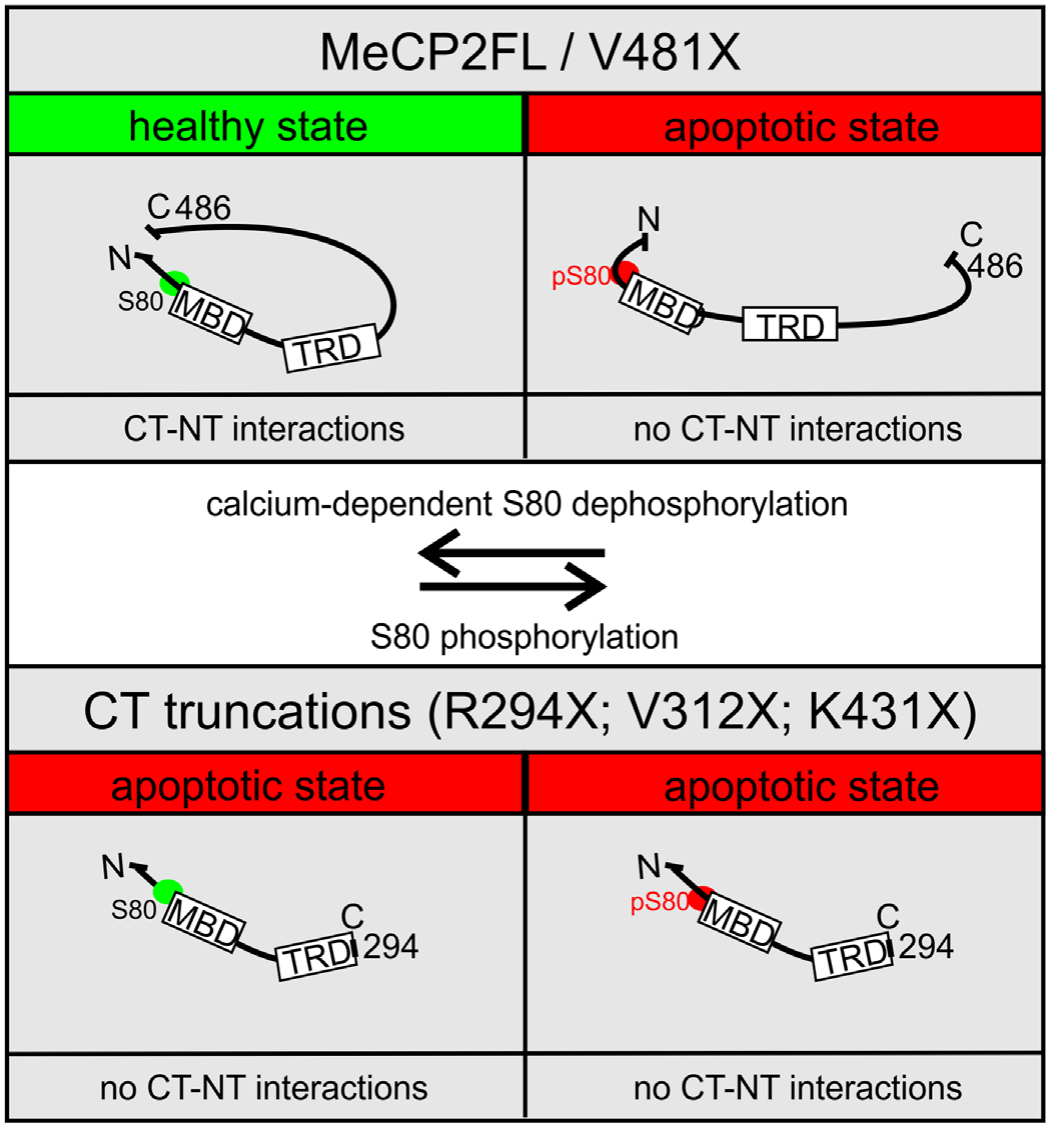
Working model. Phosphorylation of MeCP2FL at S80 induces conformational changes of MeCP2 that prevent C- and N-terminal interactions (CT-NT interactions), thus inducing conformational changes that (apoptotic state, red) underlie the apoptotic effect of MeCP2 *in vivo*. Calcium dependent de-phosphorylation at S80 returns MeCP2 to a healthy conformation (green) that allows CT-NT interactions. C-terminal truncation prevents CT-NT interactions thus favoring the apoptotic conformation of MeCP2. R249X is shown as an example, but it is expected that V312X and K431X truncations act in the same manner. The short truncation V481X removes just 5 amino acids, which does not prevent CT-NT interactions thus favoring the healthy conformation of MeCP2.

While the mechanism by which MeCP2 C-terminal truncations cause apoptosis requires further investigation, we propose two possible explanations as to how this may occur. First, MeCP2 truncations, such as R294X, could take on new, divergent functions to initiate apoptosis pathways *in vivo*, such as binding to other proteins or DNA not normally influenced by MeCP2FL. The dominant negative effect that we find for *R294X* when co-expressed with *MECP2FL* (Fig 1e) may support this view. By contrast, the fact that increased expression of *MECP2FL* in cell culture causes cell death, albeit to a lesser extent than C-terminal truncated variants (Fig 2), [22, 23], suggests that it is unlikely to be an effect of a novel divergent function of the truncated protein. Alternatively, we propose that the C-terminus may instead be critical for mediating healthy functions of MeCP2 via internal interactions with the N-terminus (Fig 6).

In support of the latter, previous *in vitro* studies have shown that phosphorylation at S80 is required for apoptosis as caused by full-length MeCP2 [23]. This is in accordance to our finding that a *MECP2* transgene with a phosphomimicking mutation at S80 (*MECP2FLS80E*) also increases caspase activity in *Drosophila* motoneurons. However, we further find that C-terminal truncations cause apoptosis independently of S80 phosphorylation status. This suggests that the effects of S80 phosphorylation may be upstream to the consequences of C-terminal truncation. This is consistent with a recent report on the role of the CTD in microRNA processing [21]. This study found that S80 phosphorylation mediates internal N-C terminal interactions of MeCP2. While N-C terminal interactions are increased by activity dependent de-phosphorylation of S80, they are decreased by constitutive S80 phosphorylation, and this in turn affects the ability of the CTD to bind other interacting proteins [21].

Our data are consistent with a model in which activity induced de-phosphorylation at S80 facilitates MeCP2 N-C terminal interactions, which in turn mediate a “healthy” conformation of MeCP2 promoting cell survival. Upon S80 phosphorylation, which is promoted in the absence of activity [58], N-C interactions are inhibited and the open MeCP2 conformation activates apoptosis pathways (Fig 6). N-C terminal interactions are also eliminated by C-terminal truncation of MeCP2; thus, truncated variants remain in the “open” conformation regardless of S80 phosphorylation status (Fig 6). Intriguingly, FoxG1 binds with highest affinity to the N-terminal end of the MeCP2-E2 isoform and elimination of the FoxG1 binding region also enhances apoptosis with *MECP2* overexpression in primary neuron culture [22]. Thus, it is possible that N-C terminal interactions facilitate the binding of FoxG1 (or *Drosophila slp1*) to MeCP2, which in turn acts to suppress MeCP2 neurotoxicity. However, further experiments are needed to test whether internal MeCP2 N-C-terminal interactions promote cell survival.

### MeCP2, apoptosis, and disease

It remains an important question if and how the apoptotic function of MeCP2 contributes to the pathophysiology of Rett syndrome and other related neurodevelopmental disorders. Mutations such as *R294X* at, near, or beyond the end of the TRD, are seen with high frequency in Rett patients [45]. It is not yet determined whether a protein product is made in patients with C-terminal truncating mutations or whether the transcript is eliminated by nonsense-mediated decay; however, we and others find consistent truncated protein products when expressing human *R294X in vitro* (Fig 2) [72]. Furthermore, a truncated protein product is observed in a mouse model expressing the truncated variant *Mecp2*^308^, which exhibits symptoms recapitulating many features of Rett syndrome albeit to a lesser extent than *Mecp2* knock-out mice [73, 74]. In human patients, C-terminal truncating mutations are also associated with less severe symptoms compared to mutations in other domains [4, 29–31] which is somewhat contradictory to our findings that C-terminal truncations strongly increase the likelihood of apoptosis in neurons and HEK293T cells.

Though speculative at present, an intriguing explanation for this discrepancy may be that neurons with C-terminal truncated variants of MeCP2 may undergo apoptosis early in development. This would increase the proportion of neurons expressing the healthy *MECP2* variant and potentially improve disease prognosis. Because *MECP2* is located on the X-chromosome, most patients carry a healthy *MECP2* allele along with the mutated variant. In most Rett patients, the X chromosome follows random inactivation patterns and the mutated allele is expressed in an approximately equal number of cells within the brain to the healthy allele [75–77]. Nonetheless, there are reports of non-random X inactivation in Rett Syndrome patients. The majority of the mutations are either truncating or missense mutations disrupting the TRD or C-terminus [77–79]. Although non-random X-inactivation has been suggested to potentially improve disease prognosis in asymptomatic or mildly symptomatic female carriers [80–82], the underlying mechanisms are unknown. As an alternative explanation to non-random X-inactivation, we suggest that high rates of apoptosis of neurons with truncating mutations during early development may be the cause for improved disease prognosis. Consistent with this hypothesis and the established finding that increased *MECP2* expression increases the likelihood of apoptosis, disproportionally high numbers of leukocytes expressing the healthy X-chromosome is standard in females carrying *MECP2* duplications [3, 83]. However, it remains to be determined whether the X-inactivation patterning is similar in other tissue types, including neurons.

In mouse models, female mice heterozygous for the *Mecp2*^308^ truncated allele show a high degree of skew towards expression of the healthy *Mecp2* variant, which is correlated with severity of behavioral phenotypes [74]. Primary neuron cultures taken from these mice support the idea that this may be due to apoptosis as caused by the truncated variant; the proportion of cultured neurons expressing the healthy *Mecp2* allele over *Mecp2*^308^ is increased over time, even when initially plated equally [74]. Further experiments examining apoptosis in early neural development with *Mecp2*^308^ and *Mecp2*^TG^ overexpression mice are needed to conclusively test this hypothesis *in vitro* and *in vivo*.

## Supporting Information

S1 Fig Timeline of apoptosis in MN5 with *R294X* expression. a-d. Representative images of cPARP reactivity in MN5s at various pupal stages. C380-GAL4 driven expression of transgenes begins at early P5, but no caspase activity was observed up to stage P10. Apoptosis appears to begin between stages P10-P12, but timing is variable between preparations. MN5 somata were completely missing as early as P12 and were always completely gone 24 hours after pupal eclosion. Scale bar depicts 10 μm.
(TIF)

S2 Fig New *MECP2* transgene products localize to the nucleus and are expressed at relatively similar levels. a. Representative images of *Drosophila* MN5s (from pupal stages P8-P15) expressing *MECP2FL* or C-terminal truncated alleles. Transgenic flies were generated using phiC31 site-specific integration into the attp2 landing site to control for possible positional effects on transgene expression. MeCP2 expression was confirmed by immunohistochemistry with an antibody towards the N-terminus of human MeCP2. Nuclear location and localization of MeCP2 was confirmed by comparison with numerous images with nuclear labeling collected previously, including those previously published [54]. b. Representative Western blot and densitometry analysis of normalized relative MeCP2 levels in fly brain following pan neuronal expression of *MECP2FL* or the *K431X* truncation. No significant differences were detected (Student’s t-test). N = 4/group, each N consists of ten pooled fly heads. Scale bar depicts 10 μm. Error bars show mean +/- SEM.
(TIF)

S3 Fig FOXG1/slp1 sequence alignment. Alignment of the DNA binding domain of *FOXG1* (human and mouse) with *Drosophila* ortholog *slp1* shows high conservation in this sequence. The MeCP2 binding region (234–254 of mouse *Foxg1*) as determined by (2) is outlined in black.
(TIF)
