# Supplementary figures and images for: Apoptotic Activity of MeCP2 Is Enhanced by C-Terminal Truncating Mutations
Source: PLoS One. 2016 Jul 21;11(7):e0159632. doi: 10.1371/journal.pone.0159632 (PMC4956225; doi:10.1371/journal.pone.0159632)

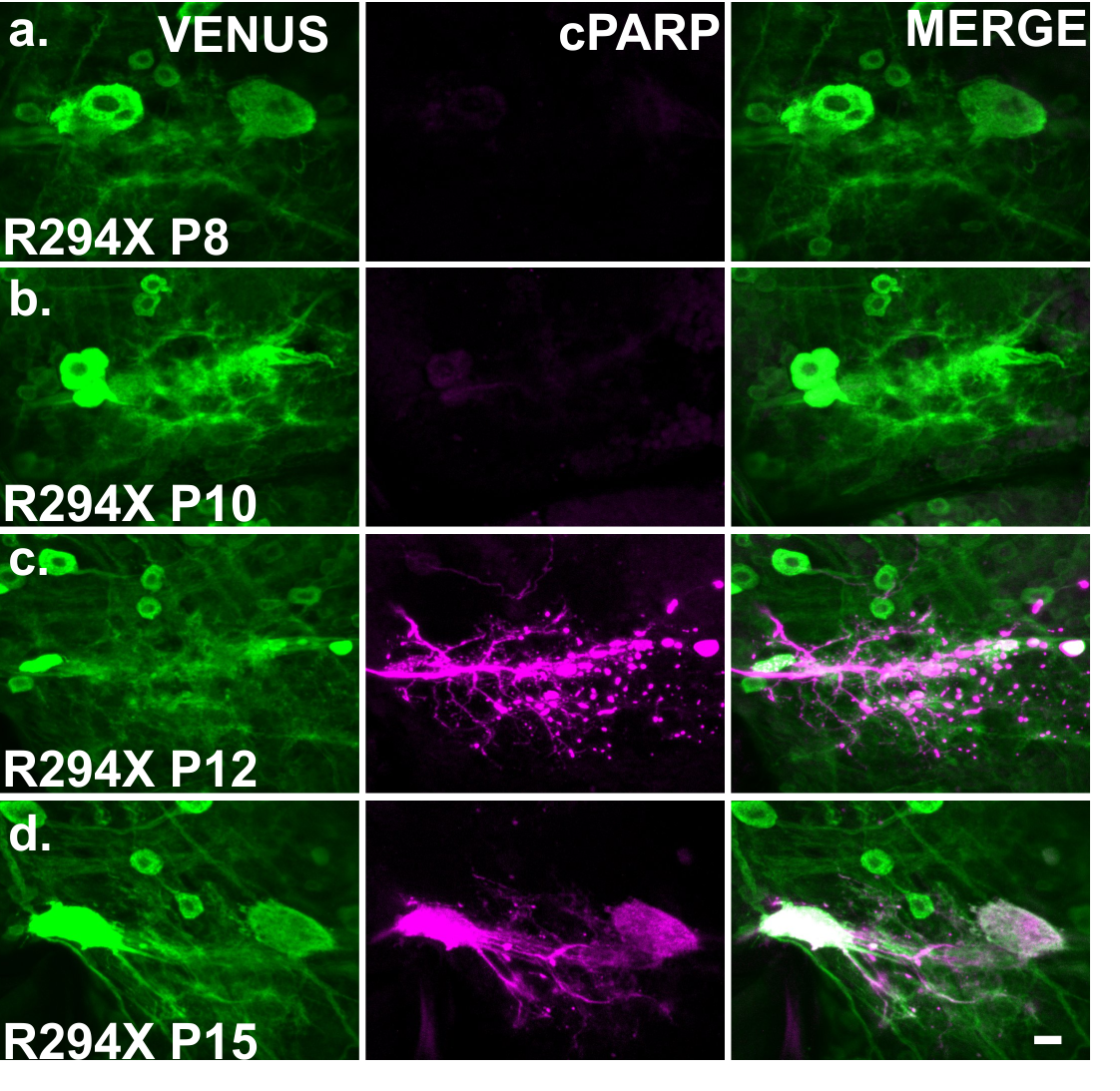

Supplement: S1 Fig — a-d. Representative images of cPARP reactivity in MN5s at various pupal stages. C380-GAL4 driven expression of transgenes begins at early P5, but no caspase activity was observed up to stage P10. Apoptosis appears to begin between stages P10-P12, but timing is variable between preparations. MN5 somata were completely missing as early as P12 and were always completely gone 24 hours after pupal eclosion. Scale bar depicts 10 μm. (TIF) [file pone.0159632.s001.tif]

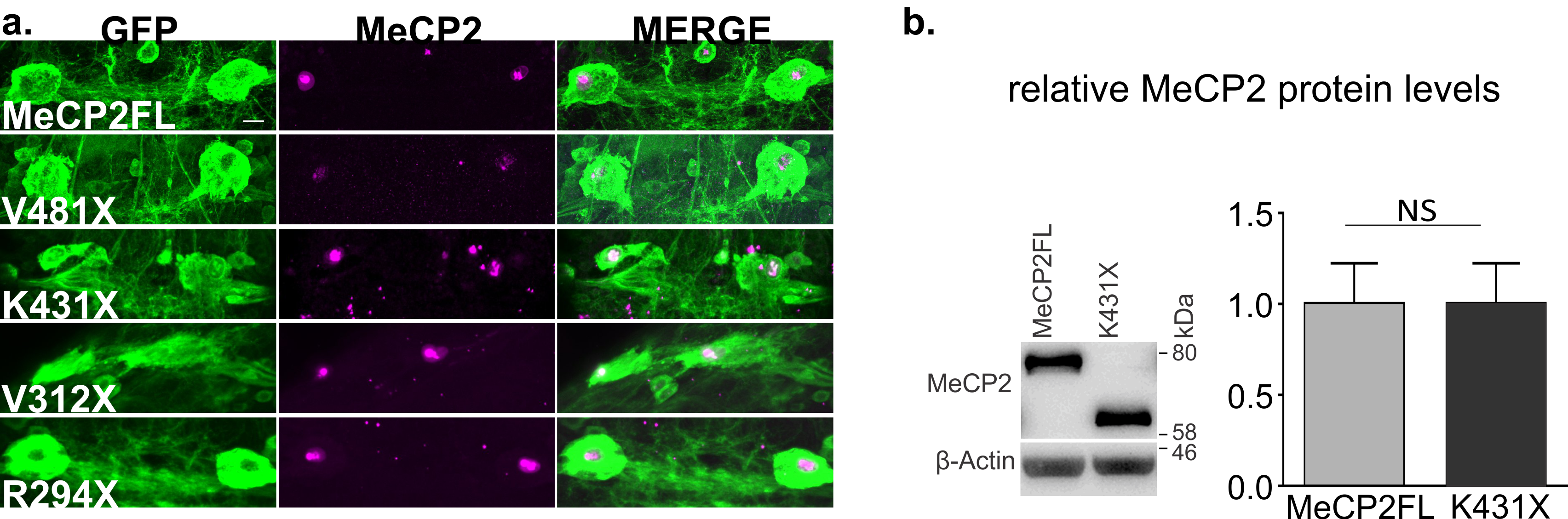

Supplement: S2 Fig — a. Representative images of Drosophila MN5s (from pupal stages P8-P15) expressing MECP2FL or C-terminal truncated alleles. Transgenic flies were generated using phiC31 site-specific integration into the attp2 landing site to control for possible positional effects on transgene expression. MeCP2 expression was confirmed by immunohistochemistry with an antibody towards the N-terminus of human MeCP2. Nuclear location and localization of MeCP2 was confirmed by comparison with numerous images with nuclear labeling collected previously, including those previously published [54]. b. Representative Western blot and densitometry analysis of normalized relative MeCP2 levels in fly brain following pan neuronal expression of MECP2FL or the K431X truncation. No significant differences were detected (Student’s t-test). N = 4/group, each N consists of ten pooled fly heads. Scale bar depicts 10 μm. Error bars show mean +/- SEM. (TIF) [file pone.0159632.s002.tif]

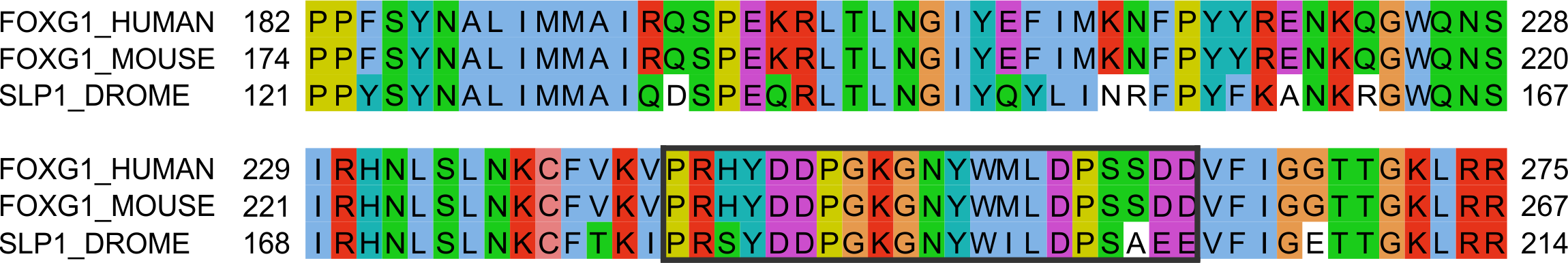

Supplement: S3 Fig — Alignment of the DNA binding domain of FOXG1 (human and mouse) with Drosophila ortholog slp1 shows high conservation in this sequence. The MeCP2 binding region (234–254 of mouse Foxg1) as determined by (2) is outlined in black. (TIF) [file pone.0159632.s003.tif]
